# Comprehensive analysis of knee cysts: diagnosis and treatment

**DOI:** 10.1186/s43019-025-00269-2

**Published:** 2025-05-14

**Authors:** Mahmod Hasan, Yaron Berkovich, Bilal Sarhan, Yaniv Steinfeld, Eyal Ginesin, Sobhe Hijaze, Ali Sleiman, Yaniv Yonai

**Affiliations:** 1 Department of Orthopedic Surgery at Carmel Hospital in Haifa, P.O. Box 2166, 0030012 Haifa, I’billin Israel; 2 Department of Orthopedic Surgery at Carmel Hospital in Haifa, Technion Faculty of Medicine in Haifa, Haifa, Israel; 3 Tel-aviv University, Tel Aviv-Yafo, Israel; 4 Department of Orthopedics Surgery at Carmel Hospital in Haifa, Haifa, Israel; 5 Present Address: Arcadia University, Glenside, PA USA

**Keywords:** Knee cysts, Popliteal cyst, Meniscal cyst, Proximal tibiofibular joint cyst, Joint pathology

## Abstract

Knee cysts are a common finding in orthopedic practice, with diagnoses that range from benign fluid collections to more complex lesions requiring intervention. This comprehensive review explores the types of cysts around the knee, including popliteal (Baker's) cysts, meniscal cysts, proximal tibiofibular joint cysts, and ganglion cysts within the cruciate ligaments. The review highlights the mechanisms of formation, clinical presentations, diagnostic methods, differential diagnoses, and treatments for each cyst type. Imaging, particularly MRI, plays a critical role in accurate diagnosis, helping differentiate cysts from other pathologies, such as tumors and vascular lesions. Treatment options vary, from conservative management for asymptomatic cases to surgical interventions, such as arthroscopic cyst removal, for symptomatic cysts or those associated with intra-articular pathologies. Emerging biological treatments, including platelet-rich plasma (PRP) and mesenchymal stem cell (MSC) therapies, show promise for addressing underlying joint degeneration and inflammation associated with certain cysts, particularly those linked to osteoarthritis. This review underscores the importance of tailored, evidence-based approaches in managing knee cysts to optimize patient outcomes. Keywords: Knee Cysts, Popliteal Cyst, Meniscal Cyst, Proximal Tibiofibular Joint Cyst, Joint Pathology

## Introduction

The knee is frequently assessed in orthopedic practice, with cystic masses often identified during exams or imaging. Accurate differential diagnosis is vital, as these fluid collections can range from benign to problematic conditions [1]. Factors such as cyst location, size, and relation to surrounding structures guide treatment planning [2]. Common knee cysts include popliteal (Baker's) cysts, meniscal cysts, proximal tibiofibular joint cysts, and cruciate ligament ganglion cysts [3, 4]. Popliteal cysts are often linked to osteoarthritis, rheumatoid arthritis, or meniscal tears, emphasizing the need to identify underlying joint pathologies [5]. MRI is crucial for diagnosing these lesions and evaluating joint communication and nearby structures [6, 7]. A thorough evaluation ensures appropriate management, whether conservative or surgical [8].

Recent studies indicate that mesenchymal stem cells (MSCs) and platelet-rich plasma (PRP) may aid in articular cartilage repair, offering potential benefits for knee cartilage degeneration and associated cysts, as these are linked to osteoarthritis pathology [9]. Knee osteoarthritis (OA) is now recognized as a joint-wide disease involving cartilage degeneration, inflammatory synovitis, subchondral bone remodeling (including bone cysts), and meniscal damage [10]. While PRP is effective in treating knee OA, its short-term benefits are limited, particularly in older patients. Combining PRP with MSCs, which promote bone healing and immune modulation, may enhance outcomes [11]. The literature on knee cyst treatments, especially biological approaches, remains sparse. This review aims to summarize existing studies on cysts around the knee.

## Methods

**Fig. 1:**
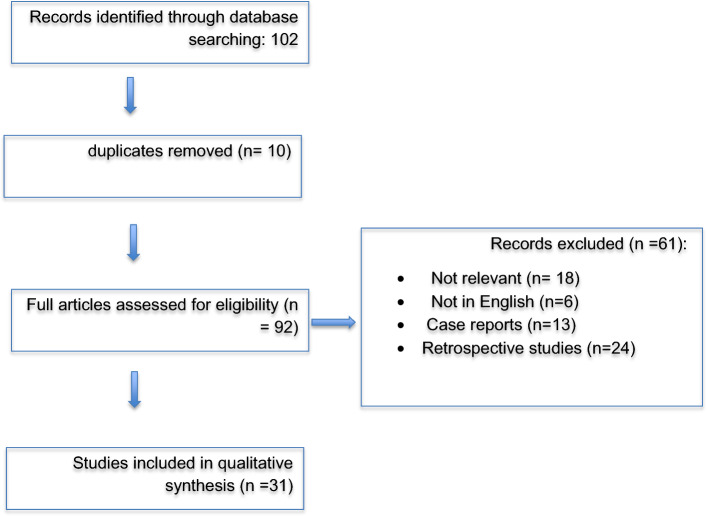
PRISMA flowchart illustrating the study selection process. The diagram outlines the identification, screening, eligibility assessment, and final inclusion of studies in the review. This structured approach ensures transparency and reproducibility in systematic reviews [4]

This systematic review was conducted in accordance with the Preferred Reporting Items for Systematic Reviews and Meta-Analyses (PRISMA) guidelines (See Fig. 1), ensuring a structured and transparent approach to literature selection. A comprehensive literature search was performed using PubMed and Embase, covering studies published in the last 25 years (1999–2024). The search included the following keywords and Medical Subject Headings (MeSH) terms: "knee cysts," "popliteal cyst", "meniscal cyst", "proximal tibiofibular joint cyst", "ganglion cyst", "subchondral cyst", "biological treatments", "PRP", "platelet-rich plasma", and "stem cell therapy". The inclusion criteria were: studies published in peer-reviewed journals, clinical studies (randomized controlled trials, cohort studies) evaluating diagnostic approaches, pathophysiology, and treatment of knee cysts, articles assessing biological treatments such as PRP, PRFM, MSC therapy, and their impact on knee cysts, and studies focusing on both conservative and surgical management. The exclusion criteria were: studies published before 1999, retrospective case reports and expert opinions without clinical data, case reports, and non-English articles that lacked an available translation.

Following the application of these criteria, 31 studies were included in the review. A PRISMA flowchart detailing the selection process is presented in Fig. 1. Data extraction was performed independently by two reviewers and cross-verified to ensure accuracy. Extracted variables included study characteristics (design, sample size, follow-up duration), cyst classification (popliteal, meniscal, PTFJ, ganglion, subchondral), diagnostic approach (MRI, ultrasound, arthroscopy), treatment strategy (nonoperative vs. surgical), biological therapies (PRP combined with hyaluronic acid (PRP + HA), PRFM, MSC therapy), and outcomes (pain relief, recurrence, functional improvement). Discrepancies between reviewers were resolved by discussion and, if necessary, consultation with a third reviewer. To ensure methodological rigor, studies were assessed using the Cochrane Risk of Bias Tool for randomized controlled trials, the Newcastle–Ottawa Scale for cohort studies, and the Jadad Score for clinical trials, ensuring the inclusion of high-quality evidence in the review.

### Popliteal cyst

**Fig. 2:**
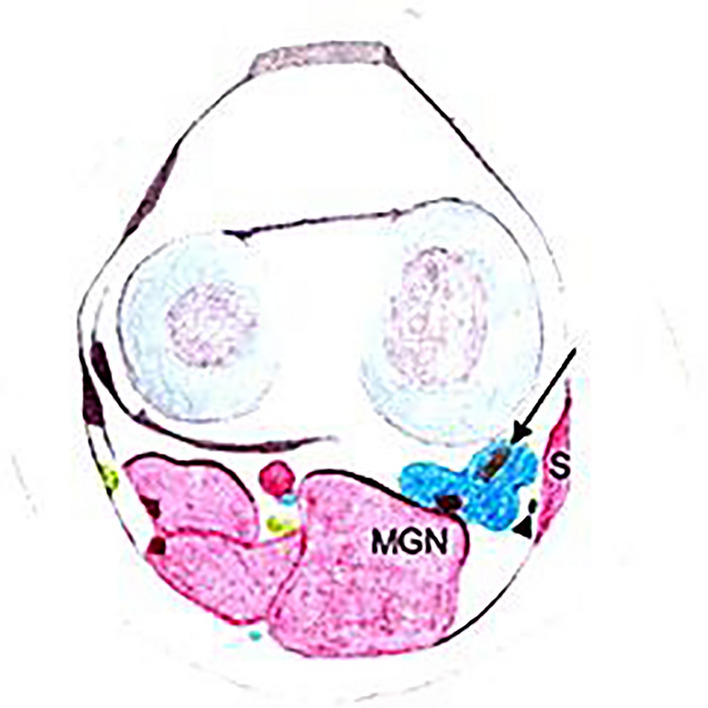
Drawing illustrating a semimembranosus- MCL bursitis. This axial view demonstrates a fluid collection in the semimembranosus-MCL bursa (blue). The semimembranosus tendon is seen passing through the fluid collection (arrow). MGN-median gastrocnemius muscle, S- sartorius muscle, arrowhead-gracilis tendon [4]

A popliteal cyst, or Baker's cyst, is a fluid-filled expansion of the gastrocnemius-semimembranosus bursa, situated beneath the medial head of the gastrocnemius muscle and the semimembranosus tendon in the medial popliteal fossa [1, 2] (Fig. 2).

The popliteal fossa contains six bursae that reduce friction between tendons, muscles, and bones. When these bursae accumulate excess fluid, cysts may form [1]. The gastrocnemius-semimembranosus bursa is the most involved in Baker's cyst formation [1, 2, 12] and communicates with the knee joint via an opening in the joint capsule located posterior to the medial femoral condyle [2].

In adults, the mechanism of cyst formation is not fully understood, but two main theories exist [1, 2]. The first theory attributes cyst formation to increased intra-articular pressure caused by conditions such as osteoarthritis, meniscal tears, or rheumatoid arthritis [1, 2]. This pressure forces fluid into a communicating bursa or leads to joint capsule herniation, with a valve-like mechanism allowing fluid to enter but not return, causing cyst accumulation [2, 12, 13]. Over time, this results in cyst development, especially in cases with effusion, inflammation, or degenerative knee diseases [2, 13] (Table 1). The second theory suggests that joint mechanics are altered by intra-articular issues, driving synovial fluid into the bursae [1]. Increased intra-articular pressure and mechanical changes push fluid into the gastrocnemius-semimembranosus bursa, where a valve-like mechanism traps it, resulting in cyst formation [2].

**Table 1:** Mechanisms of Knee Cyst Formation

Cyst type	Proposed mechanisms	References
Popliteal Cyst	Increased intra-articular pressure, joint capsule herniation, valve-like mechanism trapping synovial fluid	[1, 2, 12, 13]
Meniscal Cyst	Synovial fluid inflow through meniscal tears, myxoid degeneration	[1, 21, 23, 25]
PTFJ Cyst	Joint capsule outpouching, chronic knee effusions, bursal irritation	[1, 4, 7]
Ganglion Cyst	Mucoid degeneration of collagen structures, mesenchymal hyperplasia, synovial herniation	[1, 7, 26]
All	Biological Factors in Cyst Formation: Chronic inflammation and impaired healing response contribute to cyst persistence and recurrence. PRP + HA may help modulate this process	[30]

### Clinical presentation

Most popliteal cysts are asymptomatic and discovered incidentally during imaging for other knee issues [1, 13]. Asymptomatic Baker's cysts are reported in 4.7% to 37% of adult cases [2].

In children, the prevalence is approximately 6.3%, particularly in those aged 4 to 8 years [2, 14]. Pediatric cysts usually resolve spontaneously within 12–24 months [14] and are rarely associated with intra-articular pathologies, unlike adult cases linked to conditions such as meniscal tears or arthritis [1, 2, 13, 14]. Rare associations with osteochondritis dissecans or meniscal tears in children have been documented [2, 13].

In adults, cyst formation is attributed to openings in the joint capsule allowing one-way synovial fluid flow. This mechanism becomes more common with age due to capsule degeneration and inflammatory conditions like osteoarthritis [2, 13].

Pediatric cysts are typically asymptomatic and identified during physical exams [1, 2, 13]. When symptomatic, they may present as painless swelling behind the knee [2]. Larger cysts can cause posterior knee pain, limited flexion, or flexion contracture due to pressure on surrounding structures [14]. They are more noticeable during knee extension and can be differentiated from solid tumors using transillumination [14].

The literature on pediatric popliteal cysts is limited, emphasizing the need for further research to understand their impact and treatment outcomes.

In adults, Baker's cysts often present with swelling, pain, stiffness, and tightness behind the knee, which worsen with activity [1, 2, 13]. Occasionally, they can lead to peripheral neuropathy or ischemia [15]. Most cases are linked to intra-articular disorders, with osteoarthritis (50.6%), rheumatoid arthritis (20.6%), and gout (13.9%) being the most common associations [2].

Cyst rupture may cause sudden calf pain and swelling [1, 14]. Symptoms in adults range from mild discomfort to severe pain and restricted mobility, influenced by the cyst's size and the underlying knee condition [13]. (See Table 2 for further details.)

**Table 2:** Clinical presentation of knee cysts

Cyst Type	Location	Symptoms	Potential Complications	References
Popliteal Cyst	Popliteal fossa (gastrocnemius-semimembranosus bursa)	Posterior knee pain, swelling, stiffness	Rupture, nerve compression, DVT-like symptoms	[1, 2, 13, 15]
Meniscal Cyst	Adjacent to meniscal tears (medial/lateral meniscus)	Localized pain, swelling, locking, mechanical symptoms	Bone erosion, nerve impingement	[1, 21–23, 30]
PTFJ Cyst	Proximal tibiofibular joint	Lateral knee pain, swelling, peroneal nerve involvement	Foot drop, sensory deficits, chronic pain	[1, 4, 7]
Ganglion Cyst	Intra-articular (ACL, PCL) or periarticular	Asymptomatic or knee pain with restricted ROM	Nerve compression, joint impingement	[1, 7, 26, 28]
Parameniscal Cyst	Near the meniscus	Pain, joint instability, effusion	Increased risk of recurrence, cartilage damage	[30]

### Evaluation

Physical examination for a popliteal cyst begins with the patient supine and the knee extended. A medial mass behind the knee may be palpable, changing in size and tension with knee flexion (Foucher's sign) [1].

In children, radiographs are usually normal or show a soft tissue mass, with ultrasound confirming the diagnosis. MRI provides further detail and detects rare intra-articular issues [14].

Ultrasound is a quick, cost-effective, and sensitive method for diagnosing popliteal cysts [1]. MRI is the gold standard, offering detailed visualization of cysts and associated intra-articular pathologies, though it is typically reserved for complex cases due to cost and time [1, 15]. Arthrography and CT are used in unusual cases to differentiate cysts from conditions like DVT or popliteal artery aneurysms [1].

MRI provides precise imaging of cyst size, shape, location, and communication with the knee joint [2]. It distinguishes cysts from tumors, aneurysms, and varices, showing a well-delineated mass with low T1 signal, intermediate proton density, and high T2 signal [1, 15]. While MRA with gadolinium is used for meniscal or cartilage evaluation, its role in diagnosing knee cysts remains limited and requires further study [2, 16].

Ultrasound is the most commonly used imaging method for evaluating popliteal cysts due to its wide availability, low cost, and effectiveness, even for cysts smaller than 4 mm [15]. It provides real-time guidance for procedures such as biopsies, fluid aspiration, and injections [15] and helps monitor osteoarthritis progression, as cyst presence often indicates underlying OA [17].

On ultrasound, a popliteal cyst appears as an anechoic or hypoechoic fluid-filled structure between the semimembranosus and medial head of the gastrocnemius [15]. While less sensitive to intra-articular lesions and operator-dependent, it is practical when MRI is unavailable, aiding in cyst volume assessment and surgical planning [15]. Despite variability in studies, meta-analyses confirm ultrasound as a valuable, cost-effective alternative to MRI for diagnosing Baker's cysts [15]. (See Table 3.)

**Table 3:** Diagnostic approaches for knee cysts

Diagnostic Method	Description	Cyst Type	Advantages	Disadvantages	References
Clinical Examination	Palpation, swelling, pain assessment, mobility limitation	All	Quick, cost-effective	Low specificity, may not differentiate cyst type	[1, 4, 7]
Ultrasound (US)	Non-invasive imaging for cyst visualization	All	Fast, inexpensive, guides aspiration/injections	Operator-dependent, limited in deep cysts	[1, 4, 7, 21]
MRI	Gold standard for cyst evaluation, detecting meniscal tears, cartilage damage	All	High accuracy, detailed soft tissue visualization	Expensive, time-consuming	[1, 4, 7, 21, 30]
Arthroscopy	Direct visualization inside the joint	All	High accuracy, allows simultaneous treatment	Invasive, requires anesthesia	[1, 4, 21]
US-Guided Injection	Used to confirm cyst communication with the joint and guide aspiration or PRP therapy	Meniscal Cyst	Minimally invasive, real-time imaging	Operator-dependent, limited for deeper cysts	[30]

### Differential diagnosis

The differential diagnosis of a popliteal (Baker's) cyst includes:

- Deep Vein Thrombosis (DVT): Both cause swelling, but distinguishing is critical to prevent inappropriate anticoagulation and cyst hemorrhage [1, 13, 15].
- Anatomic Variants: Normal joint capsule recesses mimic cysts but lack communication with meniscal tears [1].
- Meniscal Cysts: Related to meniscal tears and resemble popliteal cysts [2, 15].
- Ganglion Cysts: Fluid-filled structures around the knee similar to popliteal cysts [2, 15].
- Hematomas/Popliteal Vein Varices: Swelling that imaging differentiates from cysts [1, 13].
- Tumors (Benign or Malignant): Conditions like synovial sarcoma [1, 2, 15], lipoma [13, 15] and liposarcoma [1, 13, 15], and juxta-articular myxoma [1], bone, fatty, hamartoma tumors [2] Malignant Fibrous Histiocytoma [13], can resemble popliteal cysts and these should be ruled out with imaging [1, 2].
- Popliteal Artery Aneurysm: Mimics cyst with posterior mass and swelling [2, 13].
- Synovial Hemangioma/Abscess: Vascular lesions or pus collections that mimic cysts [13].

### Treatment of Baker’s Cyst (See Table 4)

**Table 4:** Treatment Strategies for Knee Cysts

Treatment Type	Description	Advantages	Disadvantages	Effectiveness	Cyst Type	References
Conservative Treatment	NSAIDs, physiotherapy, aspiration, corticosteroid injections	Minimally invasive, reduces symptoms in mild cases	High recurrence rate	Varies, limited evidence	All	[21, 23]
Arthroscopic Treatment	Meniscal tear debridement and cyst management via minimally invasive surgery	Effective, good clinical outcomes, allows meniscus preservation	Some recurrence, surgical risks	86.9% success rate	Meniscal Cyst	[21, 23]
Open Surgery	Full cyst removal via incision, often used for larger or recurrent cysts	Low recurrence (3.41%), high success rate (87.5%)	More invasive, longer recovery	High success rate	All	[22, 23]
PRP + HA Injections	Intra-articular injections combining PRP with hyaluronic acid to reduce inflammation and promote healing	Minimally invasive, potential for pain relief and cyst reduction	Limited long-term data, requires multiple injections	70% success rate in treating meniscal cysts	Meniscal Cyst	[30]
PRFM-Augmented Surgical Repair	Use of platelet-rich fibrin matrix (PRFM) to enhance healing after cyst resection	May improve healing and reduce recurrence	Experimental, no long-term data	Potential to reduce recurrence rates	Parameniscal Cyst	[29]
Ultrasound-Guided Aspiration & Injection (UGAFI)	Used for cyst decompression and simultaneous PRP injection	Minimally invasive, cost-effective	High recurrence risk if meniscal pathology is not treated	Promising but requires further study	Meniscal Cyst	[30]

#### Nonoperative treatments

- Children: Reassurance and follow-up are standard; surgery is rare, reserved for large, symptomatic cysts or restricted knee movement [14].
- Ultrasound-Guided Aspiration and Injection (UGAFI): Minimally invasive procedure involving fluid aspiration, cyst wall fenestration, and corticosteroid injection under ultrasound guidance. It effectively reduces symptoms, cyst size, and inflammation [5].
- Corticosteroid Injections: Ultrasound-guided intracystic or intra-articular injections provide short-term pain relief and reduce inflammation, often combined with treatments targeting joint pathology [8].
- Radiation Therapy: Rarely used, it can reduce cyst size and pain in refractory cases. Photon radiation decreases NRS pain scores significantly, with 43% achieving near-complete resolution (< 1 mL fluid) at 9 months [8]. Historical methods, such as radioactive gold injections, are now impractical due to high radiation doses [13].

### Operative treatments

#### Arthroscopic cyst wall resection

Is a minimally invasive technique for Baker's cyst treatment, involving cyst wall removal and addressing intra-articular lesions, such as meniscal tears [6]. It has a high success rate of 98.2%, low recurrence (1.2%), and complete cyst resolution in many cases, with follow-ups of 12.5–36.1 months [6, 18]. The procedure uses an arthroscope for cyst debridement and re-establishing joint communication, often through a posteromedial portal [6].

### Advantages

- High success rate with a 3.1% recurrence (vs. 40% in open excision) [12, 18].
- Simultaneous treatment of intra-articular pathologies improves knee outcomes [12].
- Minimally invasive, leading to faster recovery, better cosmetic results, shorter surgical time, and cost-efficiency [12, 18].
- Lower VAS pain scores and better healing rates [12].
- Improved knee function and early mobilization, reducing DVT risk [12].
- Restores joint communication and reduces infection risk with sterile saline [12].

### Disadvantages

- Higher complication rate (6.9% vs. 1.1% for cyst wall preservation) [18].
- Risk of neurovascular injury, especially with larger cysts [18].
- Potential for popliteal artery pseudoaneurysm and hematoma formation [18].

### Arthroscopic valve debridement without cystectomy

This procedure debrides the valve-like opening between the cyst and knee joint to prevent unidirectional fluid flow, using a posteromedial portal to enlarge the opening and address intra-articular pathologies such as meniscal tears. An intracystic portal may be added for complete debridement. While effective, it has a slightly higher recurrence rate than cystectomy [8, 19].

### Advantages

- High success rate with fewer complications and minimal recurrence [19].
- Minimally invasive, leading to shorter recovery times and less postoperative pain [19].

### Disadvantages

- Higher recurrence rate (8.0%) compared to cystectomy [19].
- Risk of incomplete treatment, especially in complex anatomy [8].
- Potential vascular damage, though lower than in more invasive procedures [8].

### Communication enlargement surgery

This arthroscopic procedure enlarges the opening between the cyst and knee joint to allow bidirectional fluid flow, addressing the root cause of cyst formation. It has a 96.7% success rate, with minimal recurrence, and often includes removing obstructive tissues and enhancing fluid exchange [6].

### Advantages

- High success rate among treatments for popliteal cysts [6].
- Minimally invasive with shorter recovery times [6].
- Simultaneous treatment of intra-articular pathologies improves outcomes [6].

### Disadvantages

- Low but possible recurrence if communication is not fully enlarged [6].
- Risk of incomplete treatment if channels are not properly identified [6].
- Requires expertise to avoid complications or incomplete results [6].

Communication enlargement surgery is highly effective and minimally invasive, making it an ideal treatment option [6].

### Open excision

In Children: Rarely performed, reserved for significant symptoms, with a recurrence rate of 30–40% [14].

In Adults: Involves removing the cyst via a posterior knee incision. Historically, recurrence rates were up to 63% when underlying joint issues were untreated, but addressing causes like meniscal tears lowers this risk [6]. The procedure includes an S-shaped incision, cyst excision, and reinforcement of the joint capsule and surrounding structures to minimize recurrence [8].

### Effectiveness and recurrence

Effective but more invasive, with longer recovery times compared to arthroscopy [6]. Recurrence is high unless joint pathologies are managed, though some studies report symptom relief in 29 of 41 cases [8].

### Disadvantages and complications

- High recurrence rate if joint issues aren't addressed (up to 63%) [8].
- Wound healing complications, including calf swelling that may mimic DVT [8].
- Less favorable cosmetic outcomes due to scarring [12].

While effective, open excision is less preferred due to invasiveness and recurrence risks [6, 8].

In summary, "Open excision of popliteal cysts remains an option but carries higher risks of recurrence and complications compared to less invasive methods, particularly when underlying intra-articular pathologies are not addressed.

In the context of knee osteoarthritis treatment, biological therapies such as PRGF (plasma-rich growth factors) have been applied through intraosseous infiltrations into the subchondral bone and intra-articular injections targeting cartilage and synovial tissue. These approaches aim to reduce pain and inflammation and are often performed as office-based procedures using WALANT (Wide-Awake Local Anesthesia No Tourniquet) [20].

Despite the increasing global use of PRP, PRGF, and MSC injections, their direct impact on Baker’s cysts remains unstudied. While these biological therapies have demonstrated efficacy in managing osteoarthritis and meniscal pathology, no studies have specifically investigated their effect on popliteal cysts. Further research is needed to evaluate their role in symptom relief, recurrence prevention, and potential integration into treatment algorithms for Baker’s cysts."

### Meniscal cyst

A meniscal cyst is a localized collection of synovial fluid within or near the meniscus, classified into intrameniscal (fluid within a torn or degenerated meniscus) and parameniscal (fluid leaking through a tear into surrounding tissue) types. Most tears associated with meniscal cysts are horizontal [4, 8, 18]. Prevalence ranges from 1 to 8%, with typical diagnosis in individuals aged 30–40 [21].

### In children

Meniscal cysts are rare, with most data based on adult studies. Incidence ranges from 4 to 8%, predominantly involving parameniscal cysts in the medial compartment [22].

### In adults

Meniscal cysts are more common in the lateral meniscus (72.28%) [23]. Medial cysts are usually near the posterior horn but may also involve other regions. Lateral cysts are often near the anterior horn or body, sometimes extending into the intercondylar notch or near cruciate ligaments. Differentiation from other fluid collections requires careful observation [4, 8, 24].

### Mechanism of formation (see Table 1)

**Fig. 3:**
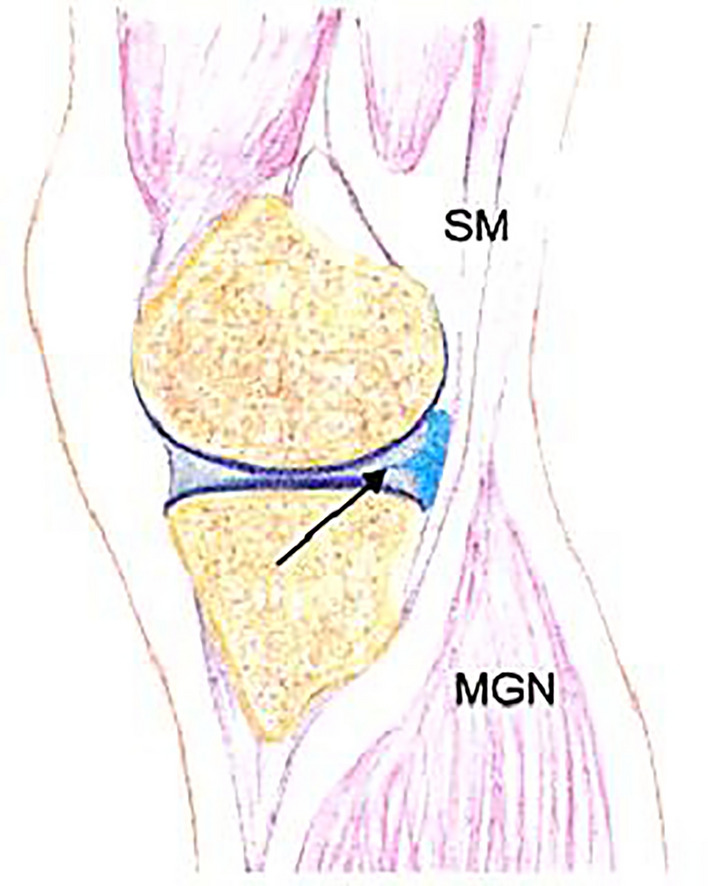
Drawing illustrating a parameniscal cyst. This sagittal view demonstrates a parameniscal cyst (blue) arising from a medical meniscal tear (arrow). SM-semimembranosus tendon, MGN- medial gastrocnemius muscle [4]

Trauma is a key factor in younger patients, while degeneration is significant in older individuals [21]. Myxoid degeneration or synovial fluid inflow through meniscal tears are primary mechanisms, often coexisting [21–23]. Horizontal tears, especially those extending to the peripheral rim, are strongly linked to cyst formation, with reports of associations in 50% to 100% of cases [21, 23, 25] (Fig. 3).

While lateral cysts were historically believed to dominate (10:1 ratio), recent MRI studies show a 2:1 ratio favoring medial cysts [23]. Repair methods like the all-inside suture technique are associated with a 29.5% cyst prevalence, though most are asymptomatic [21, 23].

### Clinical presentation

Meniscal cysts are often asymptomatic and detected incidentally on imaging but may cause activity-related knee pain, swelling, locking, popping, anterior knee instability, reduced range of motion, and other mechanical symptoms [1, 21, 22]. Severe complications include peroneal nerve palsy, tibial plateau erosion, and abnormal bone formation [21].

Case reports highlight rare presentations, such as bony erosion from a pericruciate meniscal cyst causing anterior instability and pain [1] or a lateral meniscal cyst compressing the common peroneal nerve, resulting in muscle weakness and sensory deficits [1].

### Diagnosis (See Table 3)

### Clinical examination

Meniscal cysts are often palpable, especially lateral meniscus cysts, with detection rates of 80%–100% [1, 21]. Pisani's maneuver helps differentiate meniscal cysts by observing the cyst's disappearance during knee flexion as it empties into the joint [1, 21].

### MRI

MRI is the gold standard for diagnosing meniscal cysts, providing detailed visualization of intra- and extra-articular structures, including connections to meniscal tears [1, 21].

- Intrameniscal Cysts: Appear as intermediate T2 signals within an enlarged meniscus, often linked to degeneration [22].
- Parameniscal Cysts: Present as high T2 signal fluid collections near the meniscus, often caused by horizontal tears [22, 24].

Parameniscal cysts are strong indirect indicators of meniscal tears, with a positive predictive value exceeding 90%, except near the anterior horn of the lateral meniscus, where it drops to 64% [25]. The absence of a horizontal tear on MRI usually excludes parameniscal cyst diagnosis [26].

### Ultrasound

Ultrasound is a valuable alternative for detecting meniscal cysts when MRI is unavailable, offering 97% sensitivity and 94% specificity, with 100% positive and 94% negative predictive values [1, 21]. It is noninvasive, cost-effective, and enables dynamic imaging with knee motion [1]. Recent studies provide Level A evidence supporting its effectiveness for diagnosing meniscal tears and cysts [17, 27]. However, MRI remains the gold standard due to its superior accuracy [27].

### Diagnostic arthroscopy

Diagnostic arthroscopy can identify meniscal cysts but is rarely used due to its invasiveness, limited capability for detecting extra-articular lesions, and challenges in visualizing the posteromedial meniscus [1].

### Differential diagnosis of meniscal cysts

- Normal Anatomic Variants: Capsular recesses or joint capsule extensions may mimic meniscal cysts but lack communication with meniscal tears [1].
- Bursitis: Needs differentiation from cysts near meniscal structures [22].
- Ganglion Cysts: Lack connection to the meniscus; MRI can confirm their location and absence of communication with meniscal tears, often near the PCL [1, 22, 25].
- Synovial Sarcoma: Rare, mimics meniscal cysts, and requires biopsy for diagnosis [25].
- Popliteal (Baker's) Cyst: Found near the medial gastrocnemius head, typically linked to intra-articular pathology [1, 22].
- MCL Bursitis: Appears as inflammation between MCL layers on MRI, unlike meniscal cysts, which are superficial to the ligament [1].
- Other Conditions: Includes iliotibial band friction syndrome, intra-articular pathologies, neoplasms, and soft tissue masses [21].

### Treatment of meniscal cysts

Meniscal cysts, often associated with peripheral meniscal tears, are treated by addressing the tear and decompressing the cyst to reduce recurrence risk [22, 23, 25]. Horizontal tears, common in these cases, are best managed with repair to preserve the meniscus and prevent recurrence [23]. Historically, open cystectomy and total meniscectomy caused degenerative changes, shifting current approaches toward meniscal preservation [1, 23].

### Treatment options (Table 4)

- Conservative Treatment: Aspiration with a large-bore needle and corticosteroid injection, or ultrasound-guided percutaneous decompression [21].
- Arthroscopic Treatment: Preferred for meniscal tear debridement and cyst management. Combined with partial meniscectomy or open cystectomy in severe cases, it achieves good outcomes (86.9%) but has a recurrence rate of 8.3%–10.2% [21, 23]. Arthroscopy-assisted percutaneous decompression reported no recurrences [21].
- Percutaneous Aspiration: For cysts not linked to meniscal tears, preserving meniscal tissue. Symptom relief was reported in 10 of 18 patients, with some recurrences [1, 22].
- Open Surgery: Reserved for large cysts or severe cases. It has low recurrence (3.41%) and excellent/good outcomes in 87.5% of patients [22, 23].
- Alternative Technique: Involves perforating and debriding the cyst while re-approximating the meniscus to preserve knee function [22].
- Biological Treatment: Recent studies suggest that biological therapies such as PRP + HA may offer a viable alternative for managing meniscal cysts. A prospective clinical trial involving 202 patients, including 50 with meniscal cysts, demonstrated a 70% success rate using intra-articular PRP + HA injections, reducing pain and improving joint function. These findings indicate that PRP + HA might serve as a non-invasive option to delay or avoid surgical intervention in selected patients [30].

### Outcomes and considerations

- Arthroscopic decompression is widely used but has a higher recurrence rate than open cystectomy, which shows better long-term outcomes [23].
- Clinical improvement exceeds 80% in both methods, with excellent/good results [23].
- Larger cysts and greater tear circumference increase recurrence risk [23].
- Emphasis on meniscal preservation reduces degenerative changes, ensuring better long-term outcomes [23].
- Chang et al. reported a mean VAS score reduction of − 3.3 ± 0.7 after arthroscopic treatment, with 79.3% of patients returning to sport and 85.7% experiencing minimal symptoms postoperatively [21].

### Proximal tibiofibular joint (PTFJ) cyst

PTFJ cysts are rare, with a prevalence of 0.09%–0.76% in patients undergoing MRI for knee pain, and are more common in those with chronic knee effusions [1, 4, 7]. These cysts are typically 3–5 cm but can grow up to 20 cm. While no formal classification exists, invasive types ("ganglion migrans") are categorized by the tissue they invade, such as intramuscular, intraosseous, or intraneural [1].

### Mechanism of formation

**Fig. 4:**
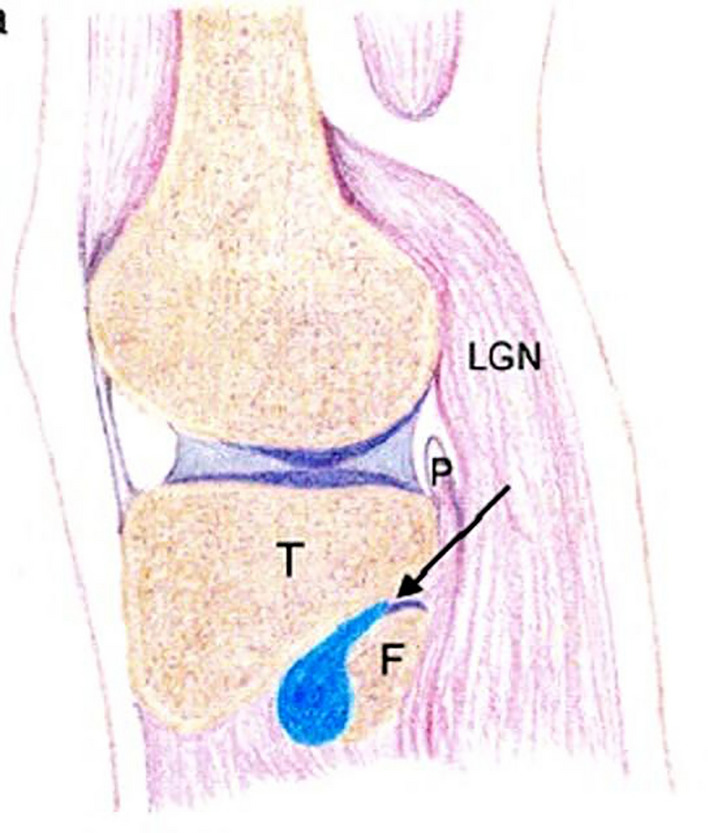
This sagittal view of the proximal tibiofibular joint (PTFJ-arrow) demonstrates a fusiform synovial cyst (blue) with a neck extending in to the PTFJ. T- lateral tibia, F- head of the fibula, P- popliteal muscle and tendon, LGN- lateral gastrocnemius muscle [4]

The etiology is unclear, but in 10% of cases, the PTFJ communicates with the knee joint. Increased intra-articular pressure may lead to joint capsule outpouching, forming synovial cysts [1, 4]. In non-communicating cysts, connections may degenerate into fibrous cords over time. Other theories include cyst development from bursae irritation, soft tissue injury, or degeneration of schwannomas or neurinomas [1] (Fig. 4). (See Table 1.)

### Clinical presentation of PTFJ Cysts

PTFJ cysts are often asymptomatic initially but can cause lateral leg pain and swelling below the knee, which worsens with activity and subsides with rest [1]. Cyst growth may lead to compression of the common peroneal nerve, resulting in motor weakness, foot drop, sensory loss along the anterolateral leg and dorsum of the foot, or compartment syndrome [1, 4]. Symptomatic cases often involve cyst hemorrhage [1]. (See Table 2).

### Differential diagnosis

- Intra-articular Degenerative Pathologies
  o Arthrosis [1]
  o Meniscal tears, particularly with joint "fullness" or lateral knee pain [1]
- Neoplasms
  o Schwannoma [1, 4]
  o Neurofibroma, presenting with similar neurologic deficits as PTFJ cysts [1, 4]
- Other Diagnoses
  o Synovial sarcoma [1, 4]
  o Juxta-articular myxoma [1, 4]

### Evaluation

MRI is the gold standard for diagnosing PTFJ cysts, providing detailed visualization of the cyst, its communication with the tibiofibular or knee joint, and its proximity to the common peroneal nerve [1, 4, 7]. It shows a homogeneous fluid lesion with a communicating neck and may detect increased muscle signal from nerve involvement, particularly on T2-weighted sequences [1, 4, 7]. Ultrasound can be used but is limited in detecting joint communication and differentiating cysts from malignancies [1]. Radiographs are often unremarkable, showing soft tissue swelling or occasional bony erosion but can detect intraosseous cysts [1]. (See Table 3).

### Management of PTFJ cysts

Symptomatic PTFJ cysts are primarily treated with surgical excision of the cyst and its joint connection, especially if the common peroneal nerve is affected, as delayed intervention reduces motor and sensory recovery chances [1]. Recurrence rates after excision range from 10 to 38%, and recurrent cysts may require re-excision or PTFJ fusion for persistent cases [1].

Image-guided aspiration with steroid injection is an option for non-neural cases but has a high recurrence rate and limited effectiveness [1, 7].

To date, no studies have evaluated the use of biological treatments such as PRP or MSC therapy for PTFJ cysts. Given their anatomical proximity to the common peroneal nerve and high recurrence rates post-excision, further research is needed to assess whether regenerative therapies could play a role in symptom management and recurrence prevention."

### Ganglion cyst

A ganglion cyst is a benign mass with a fibrous capsule, lined by spindle-shaped cells, and filled with high-viscosity mucinous fluid rich in hyaluronic acid and mucopolysaccharides [7, 28]. It typically consists of a main cyst, often lobulated with pseudopodia, and smaller capsular cysts connected to the main one. CLG cyst prevalence is 0.2%–1.9%, with a higher incidence in males [1].

### Etiology

The primary theory suggests mucoid degeneration in collagenous structures due to stress in areas like joint capsules or tendons. Repeated activity leads to tissue degeneration and gelatinous material formation. Ganglion lining cells secrete mucin-rich substances, with hyaluronic acid levels decreasing as cells degenerate [4, 7].

### Other theories

- Primary Cellular Hyperplasia: Mucin secretion causes secondary cystic degeneration; the most widely accepted theory [1, 7, 26].
- Pluripotential Mesenchymal Cell Proliferation: Mesenchymal cell activity in cruciate ligaments releases hyaluronic acid, forming cysts [1, 7].
- Synovial Herniation: Trauma or defects may cause synovial herniation, but joint connections are rare [7, 26].
- Metaplasia Theory: Proposes metaplasia from embryonic or post-traumatic remnants but lacks supporting evidence [7, 26].

### Anatomical location of ganglion cysts

Ganglion cysts commonly form near joints and can be intra-articular, extra-articular, intraosseous, or periosteal [7, 28]. Intra-articular knee ganglia are found in 0.2%–1.9% of MRI cases, with a 3:1 male-to-female ratio [7, 28].

**Fig. 5:**
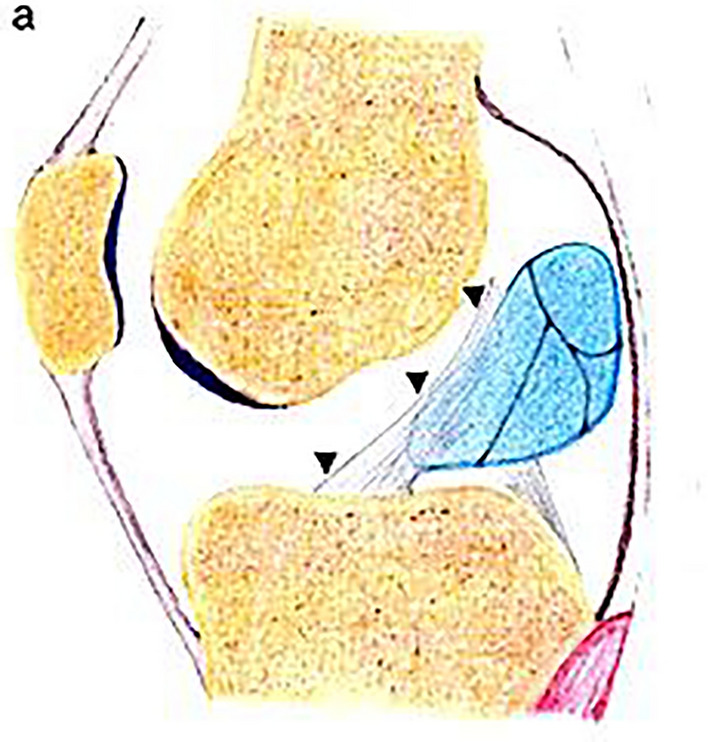
a Drawing illustrating an ACL ganglion cyst. This sagittal view demonstrates a multiloculated cyst (blue) adjacent to the fibers of the ACL (arrowheads), developing interspersed within the fibers and posteriorly to the ligament [4]

They arise in areas like tendons, ligaments, muscles, joint capsules, bursa, and nerves [26, 28]. Most intra-articular ganglion cysts are associated with the ACL, less frequently with the PCL, and occasionally in the tibiofibular joint, peroneal nerve, infrapatellar fat pad (Hoffa’s fat pad), or epiphyses of long bones near the knee [1, 7, 26, 28] (Fig. 5).

Classification is based on their position relative to cruciate ligaments (e.g., anterior to the ACL, between the ACL and PCL, or posterior to the PCL) and their size (0.5–4.5 cm) [1, 26]. Location is critical for understanding clinical presentation and guiding diagnosis [26].

### Clinical presentation of ganglion cysts (See Table 2)

- Asymptomatic Cases: Often discovered incidentally on MRI [1].
- Symptoms by Size and Location:
  o Cruciate Ligament Ganglia: Localized knee pain in areas like the medial/lateral joint line, infrapatellar, or popliteal region [1, 7].
  o Restricted Range of Motion: May present with joint locking or a palpable mass if extending extra-articularly [7].
  o Intercondylar Notch Ganglia: Worsening pain during squatting or full flexion, likely from impingement [7].
  o Ganglia in Hoffa’s Fat Pad: Can be asymptomatic or cause anterior knee pain at terminal extension with tenderness near the patellar tendon [7].
- No Trauma History: Typically, there is no history of trauma or joint instability; most intra-articular ganglia are not linked to joint derangement [7].
- Extra-Articular Ganglia: Usually asymptomatic but may cause pain or nerve symptoms, such as those affecting the common peroneal nerve [28].

### Imaging findings of ganglion cysts

#### MRI

MRI is the gold standard for CLG cyst visualization, providing detailed imaging of unilocular or multilocular cysts with sharply defined internal septa [1, 4].

- PCL Ganglia: Well-defined unilocular or multilocular cysts along the dorsal surface of the PCL [7, 28].
- ACL Ganglia: Fusiform, interspersed within ACL fibers, often extending toward Hoffa’s fat pad or the femoral intercondylar fossa. T1 images may appear isointense, resembling partial tears, but T2 hyperintensity is diagnostic [7, 28].
- Hoffa’s Fat Pad Ganglia: Well-demarcated, multilocular cysts located anterior to the anterior horn of the lateral meniscus [7, 28].
- Extra-Articular Ganglia: Appear with "bunch of grapes" pseudopodia, detectable by MRI, which also reveals muscle atrophy and fat infiltration [7, 28].

### Ultrasound (US)

#### US is useful for

- Confirming cystic fluid content [26].
- Determining cyst location and size [26].
- Identifying joint cavity communication, though this is seen in only 25% of cases [26].

US is a good initial screening tool, but MRI remains superior for detecting joint-cyst connections.

### Differential diagnosis of ganglion cysts

- Intra-Articular Ganglion Cysts: Rare (0.9%–1.3% prevalence on MRI), often originating from cruciate ligaments, Hoffa’s fat pad, or posterior septum. They may mimic meniscal cysts, causing pain, limited motion, or a palpable mass if extra-articular. Infrapatellar fat pad ganglia may cause anterior knee pain or remain asymptomatic [4, 7].
- Extra-Articular Ganglion Cysts: Found in soft tissues around the knee (tendons, ligaments, nerves), usually asymptomatic but can compress the common peroneal nerve, causing foot drop. MRI shows fluid collections with internal septations and pseudopodia, emphasizing the need to identify joint capsule connections to prevent recurrence [4].
- Periosteal Ganglia: Rarely affecting long bones, typically near the pes anserinus, resembling anserine bursitis. MRI shows sharply defined fluid masses adjacent to bone, which can mimic subperiosteal abscesses [4].
- Intra-Osseous Ganglia: Located subchondrally in long bones, they cause activity-related pain and appear as solitary cystic lesions on MRI, resembling other intra-osseous cysts and tumors [4].
- Meniscal Cysts: Originate from meniscal tears, potentially mimicking PCL ganglia if extending centrally. A communicating tear strongly suggests meniscal origin [7].
- PCL Ganglion Cysts: Typically at femoral or tibial PCL insertions, rarely surrounding the ligament [7].
- Infrahoffatic Recess: A normal fluid-filled space near Hoffa’s fat pad, visible in 13.5% of MRIs, often mistaken for ganglion cysts [7].
- Synovial Lesions: Conditions like hemangioma or synovial sarcoma may mimic ganglia but show diffuse or nodular MRI enhancement, unlike the thin peripheral enhancement of ganglion cysts [7].
- Other Pathologies: Hemangioma, synovial sarcoma, and villonodular synovitis may mimic cysts but have distinct MRI characteristics for differentiation [1].

### Management of ganglion cysts

MRI is essential for accurate diagnosis of intra-articular ganglia, as clinical detection and arthroscopy may miss some cases [7].

- Arthroscopic Excision: The preferred treatment, offering effective outcomes with no reported symptomatic recurrences [1].
- Percutaneous Aspiration: Ultrasound- or CT-guided aspiration is a cost-effective alternative, though long-term efficacy data is limited [1, 7].

Currently, there is no direct evidence supporting the use of biological therapies such as PRP or MSC injections for the treatment of intra-articular or extra-articular ganglion cysts of the knee. Given their recurrent nature and potential impact on adjacent structures, further research is needed to evaluate whether regenerative therapies could provide symptom relief and reduce recurrence rates.

### Insertional cysts

- Overview: Found in ~ 1% of knee MRI cases, insertional cysts result from bone resorption due to chronic avulsive stress at the anterior/posterior cruciate ligaments and meniscotibial attachments. They are usually asymptomatic and clinically insignificant [4, 7].
- Etiology: Chronic stress leads to focal bone necrosis, liquefaction, and cyst formation [7].
- Imaging Findings: Small, well-defined fluid-filled lesions with a thin, low-signal margin on MRI. Typically, solitary but can be multiple. Larger cysts (> 8 mm) may cause surrounding marrow signal changes [7].
- Clinical Presentation: Asymptomatic in most cases. Larger cysts may disrupt bone stress distribution, causing microfractures, edema, or hemorrhage in adjacent bone [7].

### Intraosseous ganglia

**Fig. 6:**
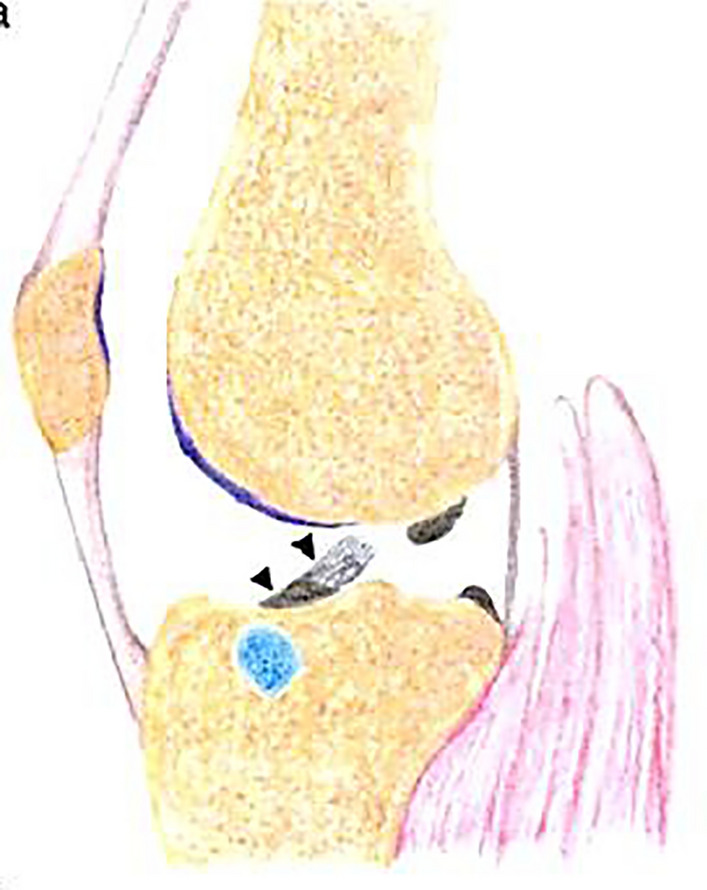
This sagittal view demonstrates an intra-osseous cyst (blue) adjacent to the insertional area of the ACL (arrowheads) at the tibia [4]

- Overview: Solitary, uni- or multilocular cystic lesions typically found in the epimetaphyseal region of long bones, especially in the tibia and subchondral bone. They lack synovial lining, joint communication, and calcification [4, 7, 28] (Fig. 6)
- Etiology: Likely caused by mucoid degeneration due to abnormal stress or parosteal ganglia extending into the bone [7].
- Clinical Presentation: The primary symptom is pain, often activity related. In some cases, pathological fractures may occur [4, 7].
- Imaging Findings: On MRI, they appear as well-defined cystic lesions near joints or ligament insertions, often with a sclerotic rim and no calcification. Joint communication may or may not be present [7, 28].
- Differential Diagnosis: Distinguished from epiphyseal bone tumors like giant cell tumor, clear cell chondrosarcoma, and chondroblastoma by:
  o Periarticular subchondral location [7].
  o Smooth, sclerotic margins [7].
  o Absence of calcification, expansion, or periosteal reaction (7).
- Management: Curettage, with or without bone grafting, is the standard treatment, with excellent outcomes [7].

### Periosteal ganglion cysts

- Overview Very rare, typically affecting the long bones of the leg, often near the pes anserinus [4, 28].
- Mechanism of Formation Caused by mucoid degeneration in the periosteum, leading to superficial bone erosion, scalloping, and reactive new bone formation. The endosteal cortex remains intact [7].
- Clinical Presentation Swelling and mild tenderness, often resembling anserine bursitis. Adjacent bone involvement is variable, with cortical remodeling, erosion, or periosteal bone formation possible [4, 28].
- Imaging Findings
  o On MRI Well-defined, homogeneous, fluid-filled masses adjacent to cortical bone. No intraosseous or discrete soft tissue components [4, 7].
- Differential Diagnosis: Includes
  o Periosteal Chondroma: Lacks internal calcification and is more common in younger patients [4, 7].
  o Cortical Desmoid [4].
  o Subperiosteal Aneurysmal Bone Cyst [4].
  o Acute Subperiosteal Hematoma: Linked to trauma or blood disorders, with similar MRI features [4, 7].
  o Subperiosteal Abscess: Involves adjacent bone marrow due to inflammation [4, 7].
  o Chronic Abscess: Appears inhomogeneous due to proteinaceous content [7].
  o Malignant Soft Tissue Tumors: Can erode bone with periosteal reaction, mimicking periosteal ganglia [7, 28].

### Subchondral cysts

**Fig. 7:**
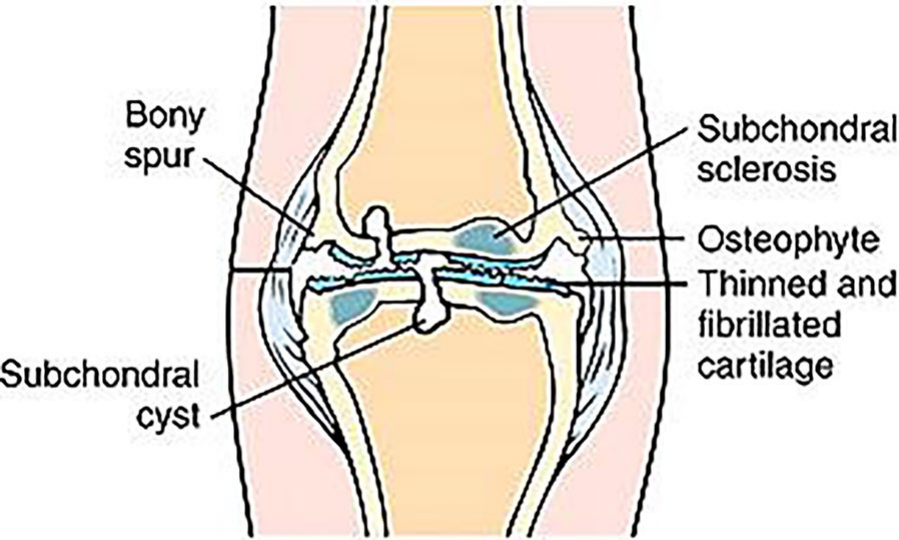
Drawing illustrating a subchondral cyst. This coronal view demonstrates a subchondral cyst in weight-bearing areas un the tibial plateau

- Overview: Also known as geodes, subchondral cysts are degenerative lesions without an epithelial lining, commonly linked to osteoarthritis [4, 28] (Fig. 7)
- Mechanism of Formation
  1. Elevated intra-articular pressure allows synovial fluid to penetrate damaged cartilage.
  2. Fractures or vascular insufficiency in the subchondral bone lead to cystic necrosis [4, 28].
- Imaging Findings
- Appear on MRI as small, multiple lesions with surrounding sclerosis, often in weight-bearing areas like the medial femoral condyle and tibial plateau [4, 28].
- Associated findings include osteophyte formation, cartilage loss, joint space narrowing, bone marrow edema, and cartilage erosion, helping to distinguish them from other cystic lesions [7, 28].
- Differential Diagnosis
- Other intraosseous cystic lesions, such as
  - Ganglia [4, 28].
  - Insertional cysts [4].
  - Intraosseous abscess [4, 7].
  - Giant cell tumor [4, 28].
  - Chondroblastoma [4, 28].
  - Chondrosarcoma [4].
  - Pigmented villonodular synovitis [7].

Imaging findings are essential for accurate differentiation [4].

Currently, no studies have explored the potential role of biological therapies such as PRP or MSC injections in the treatment of insertional, intraosseous, periosteal, or subchondral cysts of the knee. Given their association with degenerative changes and mechanical stress, further research may be warranted to evaluate whether regenerative therapies could provide symptom relief or structural benefits in selected cases.

## Discussion

Cystic lesions around the knee, such as popliteal cysts, meniscal cysts, proximal tibiofibular joint cysts, and cruciate ligament ganglion cysts, require thorough evaluation for effective management. Accurate imaging, particularly MRI, is critical for diagnosing these cysts and understanding their relationship to joint structures and underlying pathologies [1, 4]. Treatment should be individualized based on symptom severity, cyst size, and associated joint disorders [5, 6]. Conservative treatments may suffice for mild cases, but surgical or arthroscopic interventions are often necessary to address underlying issues and reduce recurrence risk [8]. A comprehensive approach combining accurate diagnosis, tailored treatment, and follow-up is key to achieving optimal outcomes.

Emerging regenerative therapies, such as bone marrow mesenchymal stem cells (MSCs) and platelet-rich plasma (PRP), show promise for osteoarthritis treatment by promoting cartilage repair and reducing inflammation [9]. PRP injections, while effective short-term, may yield better outcomes when combined with MSCs, which support bone healing and immune balance [11]. Though PRP and MSC therapies are increasingly used globally for osteoarthritis, research on their specific role in knee cyst management is limited, particularly in pediatric cases.

Recent clinical evidence supports the potential role of PRP in the treatment of meniscal cysts. A prospective study involving 202 patients, including 50 with meniscal cysts, demonstrated a 70% success rate using PRP combined with hyaluronic acid (PRP + HA) for symptom relief and functional improvement [30]. While these findings highlight PRP + HA as a non-surgical option for selected patients, further long-term studies are needed to confirm its durability and effectiveness. Additionally, novel surgical techniques incorporating platelet-rich fibrin matrix (PRFM) have been described for cystic lesions, offering a potential alternative for enhancing healing and minimizing recurrence risk [29]. These advancements underscore the growing interest in biologically-driven interventions, reinforcing the necessity for continued research into their application for knee cysts and related pathologies.

### Clinical implications of biological treatments for knee cysts

The integration of biological treatments such as platelet-rich plasma (PRP) combined with hyaluronic acid (HA) and platelet-rich fibrin matrix (PRFM) into clinical practice presents a potential shift in the management of knee cysts. Traditionally, knee cysts—particularly meniscal and popliteal cysts—have been managed with a combination of conservative measures (NSAIDs, physical therapy, aspiration) and surgical interventions (arthroscopic cystectomy, open excision). However, recurrence rates remain a concern, particularly in cysts with intra-articular communication or those associated with degenerative knee pathologies.

Recent studies suggest that PRP + HA injections may help reduce inflammation and improve joint function in patients with meniscal cysts, potentially delaying or preventing the need for surgical intervention. Similarly, PRFM has been proposed as an adjunct to surgical cyst repair, enhancing healing and reducing recurrence rates. These biological therapies aim not only to alleviate symptoms but also to address the underlying inflammatory and degenerative processes contributing to cyst formation and persistence.

Despite these promising developments, long-term data on the durability, recurrence rates, and overall efficacy of biological treatments remain limited. Current studies primarily assess symptom relief and functional improvement within a follow-up period of 6–12 months [29, 30], leaving uncertainty regarding the sustainability of these effects over multiple years. Unlike surgical excision, which provides immediate structural removal of cysts, biological therapies target inflammatory and degenerative pathways but lack definitive evidence on their ability to prevent recurrence over time.

To establish the role of PRP + HA and PRFM in standard clinical practice, future research should focus on prospective, multi-center randomized controlled trials (RCTs) with extended follow-up periods of at least 3–5 years. These studies should comprehensively evaluate recurrence rates, structural joint integrity, and functional outcomes. Additionally, further comparisons between PRP formulations, injection frequencies, and combination therapies versus surgical interventions will refine treatment protocols and provide a clearer evidence-based framework for clinical decision-making. If proven effective in the long term, biological therapies could redefine knee cyst treatment by offering less invasive and potentially more sustainable alternatives to traditional surgical approaches.

## Conclusion

Knee cysts require accurate diagnosis and individualized treatment based on their type, size, and associated joint pathology. While conservative and surgical options remain standard, emerging biological therapies like PRP + HA and PRFM show promising potential. Current evidence supports their role in symptom relief, but long-term efficacy and recurrence prevention remain unclear. Further research is needed to define their place in clinical practice.

## Data Availability

Hasan, M., Berkovich, Y., & Yonai, Y. (in press). Comprehensive Analysis of Knee Cysts: Diagnosis and treatment. *Knee Surgery & Related Research*.
